# The Every Mind Matters campaign: changes in mental health literacy and its associations with campaign awareness

**DOI:** 10.1093/eurpub/ckad145

**Published:** 2023-08-14

**Authors:** Jane S Hahn, Kia-Chong Chua, Rebecca Jones, Claire Henderson

**Affiliations:** Division of Psychiatry, Faculty of Brain Sciences, University College London, London UK; Health Service and Population Research Department, King’s College London, Institute of Psychiatry, Psychology, and Neuroscience, London, UK; Division of Psychiatry, Faculty of Brain Sciences, University College London, London UK; Health Service and Population Research Department, King’s College London, Institute of Psychiatry, Psychology, and Neuroscience, London, UK

## Abstract

**Background:**

The aim of this study is to investigate the effects on population level mental health literacy (MHL) of Every Mind Matters over 30 months following campaign launch.

**Methods:**

To observe changes in MHL over time, we conducted regression analyses on a nationally representative, repeated cross-sectional dataset of nine survey waves from September 2019 to March 2022 and an individual participant data meta-analysis with data from October 2019 to March 2021 to examine the association between campaign awareness and the outcomes.

**Results:**

There were small improvements in knowledge of management of stress, depression and anxiety, mental health vigilance, sleep literacy and psychological wellbeing self-efficacy from September 2019 to March 2020 and a deterioration in most MHL outcomes from March 2020 compared with September 2019. Campaign awareness was positively associated with symptom management of depression and anxiety, help seeking self-efficacy, stigma related to mental disorders and mental health vigilance.

**Conclusions:**

There is little evidence that the campaign improved MHL in the general population beyond March 2020. Those who were aware of the campaign may have benefitted from its resources.

## Introduction

Mental health literacy (MHL) has been defined as (i) knowledge of how to obtain and maintain positive mental health, (ii) knowledge of the symptoms and management of stress, anxiety and low mood, (iii) help seeking self-efficacy and the ability to promote one’s own mental health and (iv) stigma related to mental disorders.^1^ MHL is associated with more effective mental health practices,^2^ psychological wellbeing,^3^ and outcomes related to common mental disorders (CMDs)^4^ in the general population. According to the Adult Psychiatric Morbidity Survey, in 2014, one out of six adults in England (ages 16–65) has a CMD such as depression or anxiety.^5^ Since the last Adult Psychiatric Morbidity Survey, the COVID-19 pandemic has exacerbated multiple risks to mental health including financial insecurity,^6^ social isolation,^7^ bereavement^8^^,^^9^ domestic abuse^10^ and occupational exposures to COVID.^11^^,^^12^ Therefore, promoting MHL at population level is an increasingly important public mental health goal.

Public Health England (PHE) developed a suite of mental health digital support resources and a promotional campaign, Every Mind Matters, which launched in October 2019. Its target was to help adults to take positive actions around their mental health. The digital resources comprise of the National Health Service (NHS)-assured content covering guidance on actions that people and the public can take to address the four most common sub-clinical mental health concerns: stress, anxiety low mood and sleep problems. Amongst these digital resources was the ‘Mind Plan’, which is an online questionnaire based on the Warwick-Edinburgh Mental Wellbeing Scale,^13^ and it assesses the wellbeing of individuals and provide them with a tailored set of self-care actions to help them care for their mental health. By providing and encouraging the use of these digital resources PHE’s aim was to help prevent common mental health conditions from worsening and requiring NHS intervention.

Based on a pilot in the two Midlands regions of England in October 2018, PHE moved from a strategy that focussed on providing information about common mental health disorders to one that delivers and promotes evidence-based digital resources to facilitate self-care action for sub-clinical mental health problems for the national launch in October 2019. A second campaign under the Every Mind Matters banner across January/February 2020 encouraged people to talk openly about their mental health. A third campaign ran during April/May 2020 with a specific focus on promoting actions for people to take to care for their mental health during the first COVID-19 lockdown in response to a ministerial request. The fourth campaign in September/October 2020 changed direction to target parents rather than adults, with the aim of encouraging and supporting them to take action to look after the mental health of their children. This campaign strategy was complemented by advertising targeted directly at teenagers to help them look after their own mental health. Web analytics showed that between 7 October 2019 and 28 February 2021 the Mind Plan was completed 3 110 763 times.

Our approach to evaluation was based on the broad definition of MHL by Kutcher using the following outcomes: (i) symptom recognition and knowledge for symptom management of stress, anxiety and depression, (ii) mental health vigilance, (iii) sleep-literacy, (iv) self-efficacy and (v) stigma related to mental disorders.^1^ Mental health vigilance fits into Kutcher’s definition of MHL as vigilance leads to the use of relevant knowledge in the maintenance of every day mental health and wellbeing.^14^

Our objectives were therefore to (i) examine changes in knowledge of the symptoms and management of stress, anxiety, low mood, sleep literacy, help seeking self-efficacy and psychological wellbeing self-efficacy, stigma and mental health vigilance among adults living in England from September 2019 to March 2021 and (ii) compare these outcomes between those aware versus not aware of the campaign.

## Methods

### Data source

Members of the general population were recruited from a market research panel maintained by YouGov (https://yougov.co.uk/) to respond to an online survey prior to the launch of Every Mind Matters. This survey was repeated eight times after national launch. YouGov used quota sampling to create a sample of 20 000 respondents demographically representative of adults living in households in England.

### Measures

 

### Exposures

We investigated change in MHL over nine survey waves (September 2019 *n* = 3000, October 2019 *n* = 2000, November 2019 *n* = 2000, January 2020 *n* = 2000, March 2020 *n* = 3000, September 2020 *n* = 2000, March 2021 *n* = 2000, September 2021 *n* = 2000 and March 2022 *n* = 2000).

Campaign awareness was assessed using the question ‘Do you remember seeing this ad recently?’ Web, television or radio advertisements were shown for the October 2019, November/December 2019, January 2020, March 2020, September 2020 and March 2021 campaign bursts. Participants responding that they had seen the content or something similar were categorised as being campaign aware. Those who answered that they had not seen the campaign or did not know they were categorised as campaign unaware. We did not include September 2019, September 2021 and March 2022 as these waves did not measure campaign awareness.

### Outcomes

Symptom recognition of stress, anxiety and depression were measured by the Mental Health Literacy—Knowledge for Recognition (MHL-REC) scale. Symptom management of stress, depression and anxiety was measured by the Mental Health Literacy—Knowledge for Management (MHL-ACT) scale.^14^ The MHL-REC consists of three identical sets of nine items and asks the participants to identify whether these experiences were a symptom of stress, depression, anxiety. Respondents could choose an answer for each condition or the option ‘none of these’. The scores range from 0 to 9 for each condition. The MHL-ACT asks whether each of seven actions could help with reducing each of depression, stress and anxiety. The response format is the same as MHL-REC and scores range from 0 to 7 for each condition. Both the MHL-REC and MHL-ACT have been evaluated for construct validity.^14^

Mental health vigilance was measured using the Mental Health Vigilance scale (MHL-VIG).^14^ The MHL-VIG comprises 12 items on attitudes about maintaining mental health. The response options are ‘strongly disagree’, ‘disagree’, ‘neutral’, ‘agree’ and ‘strong agree’. Higher scores indicate a healthier attitude towards maintaining one’s mental health. MHL-VIG demonstrated good score reliability for discriminating a large range of individual differences. MHL-VIG data also demonstrated structural validity and convergent validity with constructs treated to MHL such as symptom recognition, symptom management and mental health related stigma.

We measured sleep with the Sleep Beliefs Scale (SBS).^15^ The scale includes 20 items divided into three factors: ‘sleep-incompatible behaviours’, ‘sleep–wake cycle behaviours’ and ‘thoughts and attitudes to sleep’. The total score ranges between 0 and 20. SBS shows good internal consistency of 0.71 (Cronbach’s alpha).^15^

We measured help-seeking self-efficacy using a subscale from the Mental Health Literacy Scale (MHLS)^16^ and psychological wellbeing self-efficacy using a subscale of the Self-Rated Abilities for Health Practices Scale (SRAHPS).^17^ The help-seeking subscale of the asks respondents to rate four statements of confidence in seeking help (e.g. ‘I am confident that I know where to seek information about in mental illness’). The response format is a Likert scale with 5 options (‘Strongly disagree’, ‘Disagree’, ‘Neither agree or disagree’, ‘Agree’ and ‘Strongly Agree’). MHLS shows good internal consistency of 0.87 (Cronbach’s alpha) and good test–retest reliability.^16^ The psychological wellbeing subscale from SRAHPS consists of seven items which ask how well the respondent is able to do things that promote their mental health (e.g. ‘do[ing] things to make me feel good about myself’ or ‘change things in my life to reduce stress’). Responses are ‘Not at all’, ‘A little’, ‘Somewhat’, ‘Mostly’ and ‘Completely’. The psychological wellbeing subscale of SRAHPS demonstrated high internal consistency (Cronbach’s alpha = 0.90) and moderate test–retest reliability (*r* = 0.63).^17^

We measured stigma related to mental disorders using intended behaviour items from the Reported and Intended Behaviour Scale (RIBS).^18^ These items assess desire for social distance by enquiring whether the respondent would be willing to interact with someone with mental health problems in the future in four different contexts (living with, working, living nearby and continuing a relationship). It uses a five-point Likert scale with ‘Agree strongly’, ‘Agree slightly’, ‘Neither agree or disagree’, ‘Disagree slightly’, ‘Disagree strongly’, as options. Scores from RIBS (ranging from 4 to 20) were reverse coded so that higher scores indicated less desire for social distance. YouGov also added an option for respondents to reply, ‘don’t know’, which was coded the same score as ‘Neither agree nor disagree.’ RIBS showed strong consensus validity as rated by service users, consumers and international experts in stigma. It also demonstrated good internal consistency (Cronbach’s alpha = 0.85) and moderate to substantial test–retest reliability (*r* = 0.75).

### Demographic variables

We collected demographic data on age, gender, socioeconomic status (SES) and ethnicity (see table 1). SES was categorised using the Market Research Society’s classification system into four groups, based on the occupation of the household’s chief income earner: AB = professional/managerial occupations, C1 = other non-manual occupations, C2 = skilled manual occupations and DE = semi-/unskilled manual occupations. Ethnicity was grouped into White, Mixed-White, Asian, Black and Other based on UK census categories.

**Table 1:** Demographic characteristics and campaign awareness over wave

Demographic characteristics	September 19	September/November 2019	November/December 2019	January 2020	March 2020	September 2020	March 2021	September 2021	March 2022	Total
	*N* (%)	*N* (%)	*N* (%)	*N* (%)	*N* (%)	*N* (%)	*N* (%)	*N* (%)	*N* (%)	*N* (%)
Age										
18–24	222 (7.0)	144 (6.9)	156 (7.7)	137 (6.8)	265 (9)	200 (10)	234 (11.1)	218 (10.3)	187 (9.5)	1763 (8.6)
25–34	593 (18.7)	347 (16.5)	349 (17.2)	371 (18.5)	533 (18.1)	332 (16.6)	357 (17)	361 (17)	316 (16)	3559 (17.4)
35–44	564 (17.8)	395 (18.8)	338 (16.7)	365 (18.2)	500 (16.9)	356 (17.8)	338 (16.1)	371 (17.5)	319 (16.2)	3546 (17.4)
44–54	577 (18.2)	437 (20.8)	357 (17.6)	366 (18.2)	541 (18.3)	367 (18.4)	404 (19.2)	385 (18.2)	371 (18.8)	3805 (18.6)
55+	1211 (38.2)	774 (36.9)	824 (40.7)	771 (38.4)	1113 (37.7)	740 (37.1)	767 (36.5)	786 (37.1)	776 (39.4)	7762 (38)
Gender										
Male	1467 (46.3)	956 (45.6)	979 (48.4)	937 (46.6)	1342 (45.5)	914 (45.8)	985 (46.9)	1025 (48.3)	921 (46.8)	9526 (46.6)
Female	1700 (53.7)	1141 (54.4)	1045 (51.6)	1073 (53.4)	1610 (54.5)	1081 (54.2)	1115 (53.1)	1096 (51.7)	1048 (53.2)	10 909 (53.4)
Ethnicity										
White	2736 (86.4)	1844 (87.9)	1758 (86.9)	1751 (87.1)	2596 (87.9)	1722 (86.3)	1786 (85)	1800 (84.9)	1690 (85.8)	17 683 (86.5)
Mixed white	85 (2.7)	42 (2)	56 (2.8)	64 (3.2)	87 (2.9)	51 (2.6)	60 (2.9)	58 (2.7)	37 (1.9)	540 (2.6)
Asian	193 (6.1)	129 (6.2)	122 (6)	107 (5.3)	153 (5.2)	115 (5.8)	149 (7.1)	165 (7.8)	139 (7.1)	1272 (6.2)
Black	66 (2.1)	38 (1.8)	45 (2.2)	50 (2.5)	63 (2.1)	57 (2.9)	53 (2.5)	41 (1.9)	49 (2.5)	462 (2.3)
Other	87 (2.7)	44 (2.1)	43 (2.1)	38 (1.9)	53 (1.8)	50 (2.5)	52 (2.5)	57 (2.7)	54 (2.7)	478 (2.3)
Socioeconomic status										
AB	1497 (47.3)	1014 (48.4)	806 (39.8)	931 (46.3)	1301 (44.1)	929 (46.6)	1111 (52.9)	823 (38.8)	833 (42.3)	9245 (45.2)
C1	760 (24.0)	509 (24.3)	549 (27.1)	486 (24.2)	752 (25.5)	514 (25.8)	426 (20.3)	605 (28.5)	380 (19.3)	4981 (24.4)
C2	373 (11.8)	235 (11.2)	294 (14.5)	244 (12.1)	395 (13.4)	249 (12.5)	246 (11.7)	281 (13.2)	316 (16)	2633 (12.9)
DE	537 (17)	339 (16.2)	375 (18.5)	349 (17.4)	504 (17.1)	303 (15.2)	317 (15.1)	412 (19.4)	440 (22.3)	3576 (17.5)
Campaign awareness										
Aware	N/A	906 (43.2)	808 (39.9)	720 (35.8)	1038 (35.2)	361 (18.1)	604 (27.9)	N/A	N/A	4423 (33.6)
Unaware	N/A	1191 (56.8)	1216 (60.1)	1290 (64.2)	1914 (64.8)	1634 (81.9)	1564 (72.1)	N/A	N/A	8755 (66.4)
Web visit										
Yes	N/A	88 (4.2)	86 (4.2)	63 (3.1)	48 (1.6)	53 (2.7)	99 (4.7)	N/A	N/A	437 (3.3)
No	N/A	2009 (95.8)	1938 (95.8)	1947 (96.9)	2904 (98.4)	1942 (97.3)	2001 (95.3)	N/A	N/A	12 741 (96.7)
Total	3167	2097	2024	2010	2952	1995	2100	2121	1969	20 435

Notes: Campaign awareness was not measured in the survey wave administered on September 2019.

### Every Mind Matters web resource use

This was measured according to participants’ responses to the question ‘Before taking this survey, have you visited the Every Mind Matters website?’ The participants were given a reminder about the contents including the online Mind Plan. Respondents answering ‘Yes’ were categorised as having visited the website and not having visited if they answered ‘No’ or ‘I don’t know’.

### Statistical methods

To explore how MHL outcomes changed over time (objective 1), we used multivariable linear regression models with dummy variables representing seven survey waves of data-collection to assess the effect of time on 11 MHL outcomes. We controlled for demographic variables (age, gender, ethnicity and SES).

We expected heterogeneity in the association between campaign awareness and MHL across survey waves because: (i) respondents in each wave were cross-sectional samples of the population, not the same cohort of individuals; (ii) measurement of campaign awareness in later waves included more targeted questions about Every Mind Matters materials on television radio and the web and (iii) respondents at a later wave would have had greater average exposure to the campaign due to cumulative campaign activity. Therefore, to investigate the relationship between campaign awareness and MHL outcomes (objective 2) we used individual participant data (IPD) meta-analysis to gain an overall picture from multiple waves of survey, accounting for between-study heterogeneity across survey sample and time points. We used a two-stage random effects model with restricted maximum likelihood estimation^19^ for our IPD meta-analysis. We derived the 95% confidence intervals using the Hartung–Knapp–Sidik–Jonkman approach, which produces more adequate error rates than other methods of estimation.^20^ We treated the individual survey waves as different trials. Effect estimates were generated by using a linear regression model with awareness as the exposure and the MHL variables as the outcomes, adjusting for age, gender, ethnicity and SES. We used τ2 to measure proportion of total variability due to between-study heterogeneity.

All analyses were conducted using Stata 16.

## Results

### Objective 1: change over time

There was a total of 21 131 respondents over nine waves. The final analytical sample comprised 20 435 respondents. 696 respondents were excluded due to missing data on who answered ‘prefer not to say’ on the ethnicity item. People aged over 55, those of white ethnicity and those in the professional/managerial occupation are the biggest groups within their respective demographic categories (table 1).

All the results for change over time are presented in tables 2 and 3. There were small improvements in symptom management for stress (MHL-ACT stress) and depression (MHL-ACT depression) in March 2020. Mental health vigilance (MHL-VIG) showed small improvements from September 2019 to January 2020 and March 2020. Sleep literacy (SBS) showed the most consistent and steady improvement from September 2019 to November/December 2019. There was a small improvement for psychological wellbeing efficacy to March 2020.

**Table 2:** Changes in symptom recognition and symptom management over time

Outcome variables	September/November 2019	November/December 2019	January 20	March 20	September 20	March 21	September 21	March 22
	B (95% CI)	B (95% CI)	B (95% CI)	B (95% CI)	B (95% CI)	B (95% CI)	B (95% CI)	B (95% CI)
Symptom recognition for stress (MHL-REC stress)	0.08 (−0.07, 0.22)	0.06 (−0.08, 0.21)	0.10 (−0.04, 0.25)	−0.03 (−0.16, 0.1)	−0.08 (−0.23, 0.06)	−0.08 (−0.22, 0.07)	−0.21 (−0.36, −0.07)	−0.19 (−0.34, −0.05)
Symptom recognition for depression (MHL-REC depression)	0.06 (−0.09, 0.21)	0.06 (−0.10, 0.21)	0.02 (−0.13, 0.18)	−0.04 (−0.18, 0.09)	−0.12 (−0.28, 0.03)	−0.24 (−0.40, −0.09)	−0.29 (−0.44, −0.13)	−0.30 (−0.45, −0.14)
Symptom recognition for anxiety (MHL-REC anxiety)	0.09 (−0.06, 0.24)	0.08 (−0.07, 0.23)	0.09 (−0.07, 0.24)	0.03 (−0.10, 0.17)	−0.05 (−0.20, 0.10)	−0.17 (−0.32, −0.03)	−0.24 (−0.39, −0.10)	−0.17 (−0.32, −0.02)
Symptom management for stress (MHL-ACT stress)	0.05 (−0.07, 0.18)	0.08 (−0.05, 0.21)	0.07 (−0.06, 0.20)	0.14 (0.02, 0.25)	0.01 (−0.11, 0.14)	−0.03 (−0.16, 0.09)	−0.19 (−0.31, −0.06)	−0.26 (−0.39, −0.14)
Symptom management for depression (MHL-ACT depression)	0.06 (−0.06, 0.19)	0.09 (−0.04, 0.21)	0.04 (−0.09, 0.16)	0.13 (0.01, 0.24)	−0.07 (−0.19, 0.06)	−0.12 (−0.25, −0.99)	−0.29 (−0.41, −0.17)	−0.31 (−0.44, −0.19)
Symptom management for anxiety (MHL-ACT anxiety)	0.04 (−0.09, 0.16)	0.08 (−0.05, 0.20)	0.10 (−0.03, 0.23)	0.21 (0.10, 0.33)	0.03 (−0.09, 0.16)	−0.08 (−0.20, 0.05)	−0.17 (−0.30, −0.05)	−0.20 (−0.33, −0.07)

**Table 3:** Changes in sleep literacy, help-seeking self-efficacy, psychological wellbeing self-efficacy, stigma and mental health vigilance over time

Outcome variables	September/November 2019	November/December 2019	January 20	March 20	September 20	March 21	September 21	March 22
	B (95% CI)	B (95% CI)	B (95% CI)	B (95% CI)	B (95% CI)	B (95% CI)	B (95% CI)	B (95% CI)
Sleep (SBS)	0.13 (−0.09, 0.35)	0.32 (0.09, 0.54)	0.27 (0.04, 0.49)	0.42 (0.22 to 0.62)	0.44 (0.22, 0.67)	0.10 (−0.12, 0.32)	0.01 (−0.21, 0.23)	0.12 (−0.11, 0.34)
Help-seeking self-efficacy (MHLS)	0.08 (−0.11, 0.27)	−0.005 (−0.20, 0.19)	0.14 (−0.05, 0.33)	0.09 (−0.08 to 0.26)	−0.72 (−0.91, −0.53)	−0.59 (−0.78, −0.40)	−0.94 (−1.12, −0.75)	−0.96 (−1.15, −0.77)
Psychological wellbeing self-efficacy (SRAHPS)	0.11 (−0.22, 0.44)	−0.26 (−0.59, 0.07)	−0.09 (−0.42, 0.24)	0.42 (0.12 to 0.71)	−1.04 (−1.37, −0.71)	−0.99 (−1.32, −0.67)	−1.28 (−1.61, −0.96)	−1.32 (−1.66, −0.99)
Stigma (RIBS)	−0.14 (−0.33, 0.04)	−0.02 (−0.21, 0.17)	0.05 (−0.14, 0.24)	−0.17 (−0.34, −0.99)	−0.22 (−0.41, −0.02)	−0.31 (−0.50, −0.12)	−0.37 (−0.56, −0.18)	−0.33 (−0.52, −0.14)
Mental health vigilance (MHL-VIG)	−0.10 (−0.52, 0.33)	0.39 (−0.04, 0.83)	0.58 (0.14, 1.01)	0.60 (0.21, 0.99)	0.16 (−0.28, 0.59)	0.26 (−0.17, 0.69)	−0.49 (−0.92, −0.07)	−0.54 (−0.98, −0.10)

However, we observed reversal in these changes beyond March 2020. There was a small decline for symptom recognition for depression (MHL-REC depression), symptom recognition for anxiety (MHL-REC anxiety) and symptom management for depression (MHL-ACT depression) from September 2019 to March 2021, during the 6 months of the first pandemic lockdown in the UK. Symptom management for anxiety (MHL-ACT anxiety) showed no change from September 2019 scores by March 2020, which suggests a decline in this score from March 2020. Mental health vigilance (MHL-VIG) showed a return to September 2019 levels for September 2020 and March 2021. Sleep literacy (SBS) also follows/ed this trend, wherein scores from March 2021 showed no difference compared with September 2019. Help-seeking self-efficacy (MHLS) scores demonstrated moderate decline for September 2020 and March 2021. Psychological wellbeing self-efficacy (SRAHPS) also exhibited a large decline to September 2020 and March 2021 compared with September 2019. Stigma related to mental health disorders (RIBS) showed a small decline from September 2019 to March 2020, September 2020 and March 2021.

By March 2022, all MHL outcomes except for sleep literacy had shown a decline. There was strong evidence that symptom recognition for stress, depression and anxiety has declined since September 2019. Similar declines are seen for symptom management for stress, depression and anxiety. Help-seeking self-efficacy and psychological well-being self-efficacy outcomes have also shown to decline. Finally, there was strong evidence that stigma scores declined and mental health vigilance was also lower over 30 months of the campaign.

### Objective 2: campaign awareness

The total number of participants included in the analysis was 13 178 and excludes participants from survey waves September 2019, September 2021, March 2022 as prompted awareness of the campaign was not measured in these waves (*n* = 7257). Demographic groups with relatively higher campaign awareness were the 18–24 age group, women, Black people and people in the semi-/unskilled manual occupation group from their respective categories (table 1).

Campaign awareness had varying associations with MHL outcomes (Supplementary tables S1 and S2 and figures S1–S4). Those who encountered the campaign had slightly higher scores for symptom management of depression (MHL-ACT depression) and anxiety (MHL-ACT anxiety), and mental health vigilance (MHL-VIG) compared with those who have not encountered the campaign. Those who encountered the campaign showed moderately higher help-seeking self-efficacy (MHLS) than those who have not encountered the campaign. Campaign awareness was associated with lower desire for social distance from people with mental illness (RIBS).

We observed little heterogeneity in associations between campaign awareness and symptom recognition of depression (MHL-REC depression), anxiety (MHL-REC anxiety) and stress (MHL-REC stress), symptom management for anxiety (MHL-ACT anxiety), sleep literacy (SBS) and stigma (RIBS). We did not observe any association between awareness and symptom recognition for stress, depression, anxiety, symptom management of stress and help-seeking self-efficacy.

## Discussion

In accordance with the definition of MHL from Kutcher and colleagues,^1^ these results indicate a short-lived improvement in knowledge for managing one’s own mental health problems, sleep-literacy, ability to promote one’s own mental health in the general population. We see better outcomes in knowledge of managing one’s own mental health, ability to seek help and stigma related to mental health problems for those who are aware of the campaign.

### Strengths and limitations

We cannot establish temporality between the campaign and the outcomes. With campaign awareness, we do not know whether respondents benefitted from the campaign or whether people who were more mental health literate are more likely to remember the campaign.

While quota sampling ensures that subgroups in the population are represented within the study sample, it does not randomly draw samples from the population.^21^ Therefore, the results may not be entirely generalizable to the adult population in England.

Another limitation is that the study design is a repeated cross-sectional study. Our analyses used respondents from the baseline survey wave prior to the launch of the campaign as the comparator group. Our analyses operate under the assumption that respondents from the baseline survey wave are comparable in demographic and unobserved characteristics to the respondents from subsequent survey waves. While we use quota sampling to ensure that respondents from all survey waves are nationally representative of demographics of private households in England, there may be unobserved characteristics that differ between waves other than exposure to the Every Mind Matters campaign over time. Further, the variation that we see over time may be due to standard regressions towards the mean phenomenon. Therefore, we are not able to extrapolate a causal relationship between the intervention and MHL due to the design.

Finally, the population survey design does not measure the impact of use of the digital resources as opposed to the impact of the campaign at population level, as the subsample of people who visited the campaign website or interacted with digital resources on the website are also too small in proportion within the survey. Evaluation of the digital resource itself would require pre-post measurement of outcomes for resource users and a control group.

### Implications

Our data show an increase in MHL immediately before the COVID-19 pandemic and lower levels of MHL with the progression of the pandemic. One explanation is that experience of the pandemic normalised symptoms of common mental health problems due to difficult life circumstances and reduced self-efficacy due to limited access to certain activities for wellness or healthcare. As there was a decline in health literacy between pre- and post-pandemic in Japan, especially for those in lower economic groups,^22^ this may suggest that difficult life circumstances may negatively impact general health literacy.

However, we should not expect a sudden and large effect size with public mental health campaigns over two and a half years. Consistent positive changes across stigma related to mental disorders were only seen 5 years after the launch of the Time to Change social marketing campaign,^23^ The consistency of these insights suggests an early emphasis on behaviour should be considered for any population mental health campaign.

## Supplementary Material

ckad145_Supplementary_Data

## Data Availability

The data underlying this article will be shared on reasonable request to the corresponding author. Key points There was evidence of increase in mental health literacy from the launch of the campaign to March 2020. There is little evidence that the campaign improved mental health literacy in the general population level after March 2020. Those who encountered the campaign may have benefitted from its resources.
