# Supplementary material for: The Every Mind Matters campaign: changes in mental health literacy and its associations with campaign awareness
Source: Eur J Public Health. 2023 Aug 14;33(6):1008–13. doi: 10.1093/eurpub/ckad145 (PMC10710357; doi:10.1093/eurpub/ckad145)
Supplement: ckad145_Supplementary_Data [file ckad145_supplementary_data.pdf]

Table S1: IPD meta-analysis of campaign awareness and symptom recognition and symptom management

Outcomes	Timepoint	Effect size (95% CI)	Weight	t2 (95% CI)
Symptom recognition of stress (MHL-REC stress)	Oct 2019	0.06 (-0.17 to 0.29)	17.71	0 (0 to 0.0271)
	Nov/Dec 2019	0.04 (-0.19 to 0.26)	17.76	
	Jan 2020	-0.01 (-0.25 to 0.22)	16.52	
	Mar 2020	0.07 (-0.13 to 0.27)	23.46	
	Sep 2020	-0.18 (-0.47 to 0.12)	10.64	
	Mar 2021	-0.05 (-0.3 to 0.21)	13.9	
	Overall	-0.01 (-0.18 to 0.09)	100	
Symptom recognition of depression (MHL-REC depression)	Oct 2019	-0.06 (-0.3 to 0.18)	17.59	0.018 (0 to 0.15)
	Nov/Dec 2019	-0.02 (-0.26 to 0.23)	17.38	
	Jan 2020	-0.05 (-0.3 to 0.2)	16.65	
	Mar 2020	0.22 (0.02 to 0.43)	19.66	
	Sep 2020	-0.38 (-0.69 to -0.06)	13.1	
	Mar 2021	-0.09 (-0.36 to 0.18)	15.62	
	Overall	-0.04 (-0.24 to 0.15)	100	
Symptom recognition of anxiety (MHL-REC anxiety)	Oct 2019	0.24 (0.01 to 0.48)	17.19	0.02 (0 to 0.18)
	Nov/Dec 2019	0.07 (-0.17 to 0.3)	17.32	
	Jan 2020	0.01 (-0.24 to 0.25)	16.85	
	Mar 2020	0.2 (0 to 0.4)	19.37	
	Sep 2020	-0.37 (-0.67 to -0.06)	13.63	
	Mar 2021	0.08 (-0.19 to 0.34)	15.64	
	Overall	0.06 (-0.16 to 0.27)	100	
Symptom management of stress (MHL-ACT stress)	Oct 2019	0.07 (-0.13 to 0.26)	17.88	0.01 (0 to 0.09)
	Nov/Dec 2019	0.07 (-0.13 to 0.27)	17.38	
	Jan 2020	0.18 (-0.03 to 0.39)	16.69	
	Mar 2020	0.12 (-0.05 to 0.3)	20.33	

	Sep 2020	-0.28 (-0.54 to -0.03)	12.5	
	Mar 2021	0.12 (-0.1 to 0.34)	15.21	
	Overall	0.06 (-0.09 to 0.22)	100	
Symptom management of depression (MHL-ACT depression)	Oct 2019	0.14 (-0.05 to 0.33)	18.57	0 (0 to 0.02)
	Nov/Dec 2019	0.13 (-0.07 to 0.32)	17.44	
	Jan 2020	0.14 (-0.07 to 0.34)	16.02	
	Mar 2020	0.2 (0.03 to 0.37)	23.08	
	Sep 2020	-0.05 (-0.3 to 0.21)	10.53	
	Mar 2021	0.13 (-0.09 to 0.35)	14.35	
	Overall	0.13 (0.05 to 0.21)	100	
Symptom management of anxiety (MHL-ACT anxiety)	Oct 2019	0.2 (0 to 0.39)	18.24	0.02 (0 to 0.18)
	Nov/Dec 2019	0.18 (-0.02 to 0.38)	17.15	
	Jan 2020	0.22 (0.01 to 0.43)	15.98	
	Mar 2020	0.22 (0.05 to 0.39)	24.26	
	Sep 2020	-0.11 (-0.37 to 0.15)	10.3	
	Mar 2021	0.25 (0.02 to 0.47)	14.07	
	Overall	0.18 (0.06 to 0.29)	100	

Table S2: IPD meta-analysis of campaign awareness and sleep literacy, help-seeking self-efficacy, psychological wellbeing self-efficacy, stigma, and mental health vigilance

Outcomes	Timepoint	Effect size (95% CI)	Weight	t2 (95% CI)
Sleep literacy (SBS)	Oct 2019	0.08 (-0.27 to 0.42)	17.26	0.01 (0 to 0.09)
	Nov/Dec 2019	-0.18 (-0.52 to 0.17)	17.46	
	Jan 2020	0.1 (-0.25 to 0.44)	17.15	
	Mar 2020	0.34 (0.05 to 0.64)	20.3	
	Sep 2020	-0.28 (-0.72 to 0.17)	12.63	
	Mar 2021	-0.16 (-0.55 to 0.22)	15.2	
	Overall	0.01 (-0.24 to 0.25)	100	
Help-seeking self-efficacy (MHLS)	Oct 2019	0.01 (-0.28 to 0.31)	17.49	0 (0 to 0.02)
	Nov/Dec 2019	0.25 (-0.06 to 0.56)	16.74	
	Jan 2020	0.59 (0.29 to 0.89)	17.15	
	Mar 2020	0.43 (0.18 to 0.69)	19.18	
	Sep 2020	0.72 (0.33 to 1.11)	13.4	
	Mar 2021	0.52 (0.19 to 0.84)	16.03	
	Overall	0.41 (0.15 to 0.67)	100	
Psychological wellbeing self-efficacy (SRAHPS)	Oct 2019	-0.54 (-1.06 to -0.03)	17.14	0 (0 to 0.04)
	Nov/Dec 2019	-0.03 (-0.55 to 0.5)	16.97	
	Jan 2020	0.17 (-0.37 to 0.71)	16.82	
	Mar 2020	0.58 (0.13 to 1.03)	18.15	
	Sep 2020	1.19 (0.51 to 1.87)	14.7	
	Mar 2021	0.37 (-0.2 to 0.95)	16.22	
	Overall	0.27 (-0.33 to 0.88)	100	
Stigma related to mental disorders (RIBS)	Oct 2019	0.37 (0.08 to 0.66)	18.36	0.02 (0 to 0.23)
	Nov/Dec 2019	0.32 (0.01 to 0.63)	16.57	
	Jan 2020	0.48 (0.18 to 0.78)	17.4	
	Mar 2020	0.47 (0.21 to 0.73)	23.49	

	Sep 2020	0.02 (-0.38 to 0.41)	10.14	
	Mar 2021	0.23 (-0.11 to 0.56)	14.05	
	Overall	0.35 (0.19 to 0.51)	100	
Mental health vigilance (MHV-PHE)	Oct 2019	0.71 (0.02 to 1.39)	16.09	0 (0 to 0.32)
	Nov/Dec 2019	0.54 (-0.13 to 1.2)	17.04	
	Jan 2020	1.24 (0.57 to 1.9)	17.3	
	Mar 2020	0.92 (0.36 to 1.48)	24.58	
	Sep 2020	1.15 (0.34 to 1.96)	11.52	
	Mar 2021	1.26 (0.51 to 2.01)	13.46	
	Overall	0.95 (0.64 to 1.25)	100	

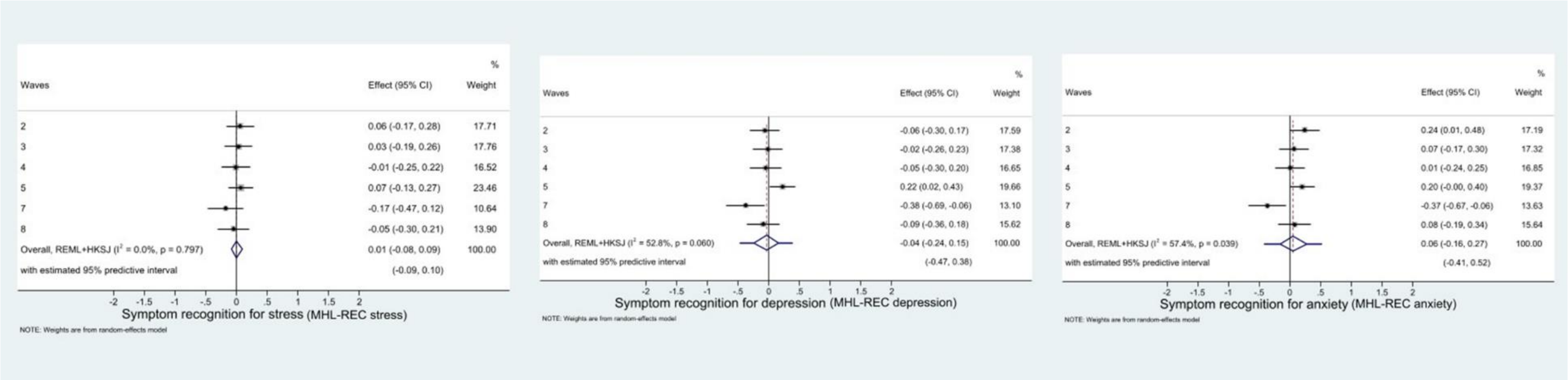

Figure S1: Effect size of awareness on symptom recognition of stress, depression, and anxiety (MHL-REC)

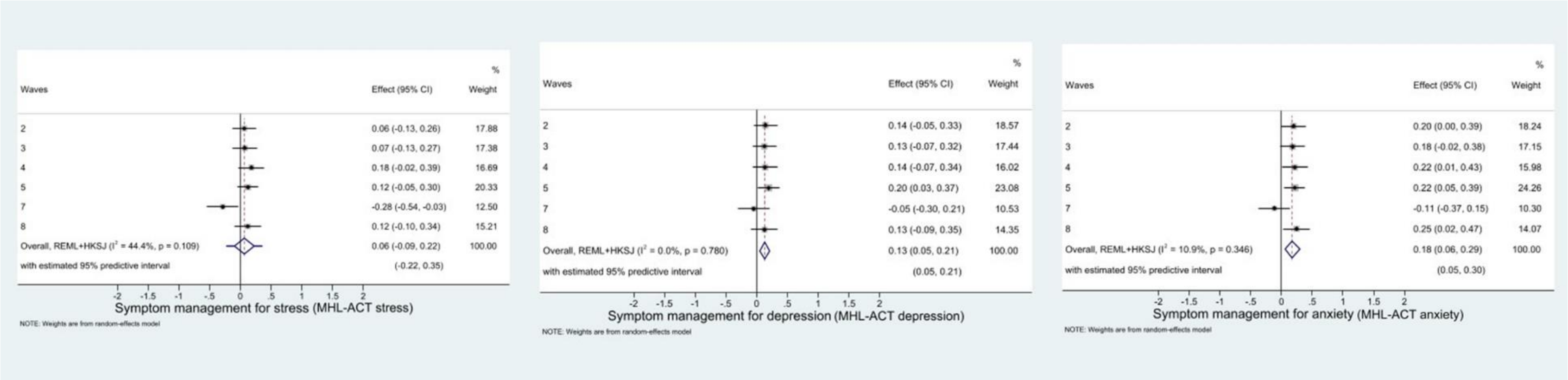

Figure S2: Effect size of awareness on symptom management of stress, depression, and anxiety (MHL-ACT)

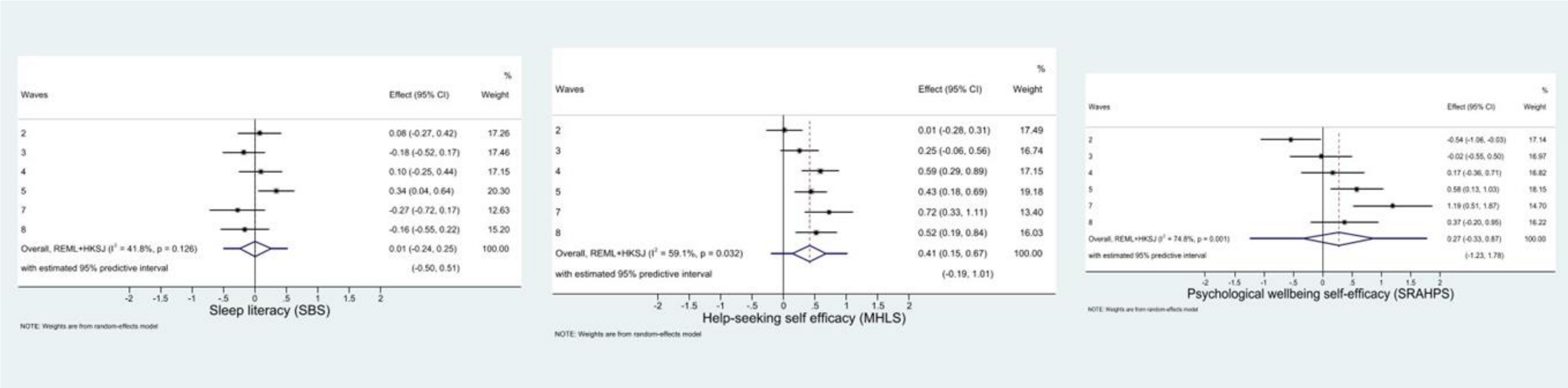

Figure S3: Effect size on sleep literacy (SBS), help-seeking self-efficacy (MHLS), and psychological wellbeing self-efficacy (SRAHPS)

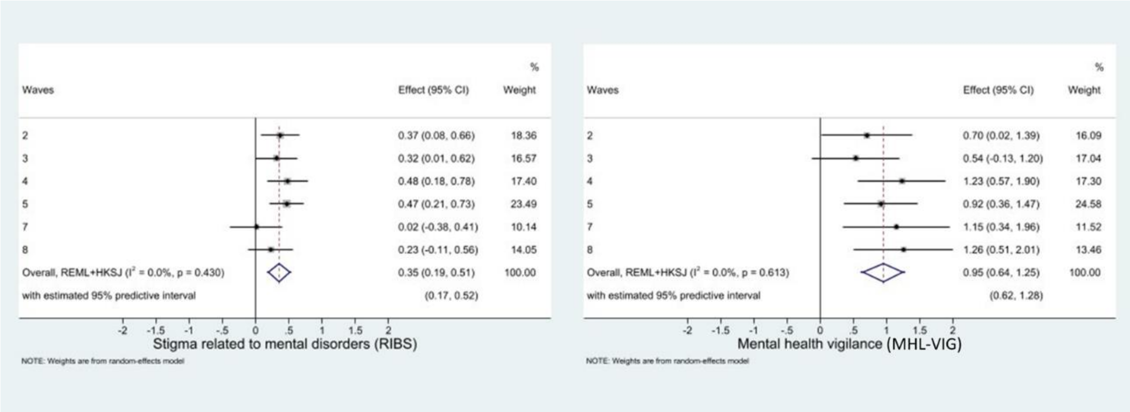

Figure S4: Effect size of awareness on stigma related to mental disorders (RIBS) and mental health vigilance (MHV-VIG)
